# Manipulation of cell migration by laserporation-induced local wounding

**DOI:** 10.1038/s41598-019-39678-1

**Published:** 2019-03-12

**Authors:** Mst. Shaela Pervin, Shigehiko Yumura

**Affiliations:** Department of Functional Molecular Biology, Graduate School of Medicine, Yamaguchi University, Yamaguchi, 753-8512 Japan

## Abstract

Living organisms employ various mechanisms to escape harm. At the cellular level, mobile cells employ movement to avoid harmful chemicals or repellents. The present study is the first to report that cells move away from the site of injury in response to local wounding. When a migrating *Dictyostelium* cell was locally wounded at its anterior region by laserporation, the cell retracted its anterior pseudopods, extended a new pseudopod at the posterior region, and migrated in the opposite direction with increasing velocity. When wounded in the posterior region, the cell did not change its polarity and moved away from the site of wounding. Since the cells repair wounds within a short period, we successfully manipulated cell migration by applying multiple wounds. Herein, we discussed the signals that contributed to the wound-induced escape behavior of *Dictyostelium* cells. Our findings provide important insights into the mechanisms by which cells establish their polarity.

## Introduction

Living organisms employ various strategies to escape risk of harm. Animals, including humans and snakes, possess the ability to avoid fires or electric shocks. Higher plants are not motile, but possess the ability to curl their leaves slant downwards^1^. In addition, chloroplasts inside plant cells can move away from the cell surface to the side when exposed to high-intensity light^2^. At the cellular level, mobile cells avoid harmful chemicals or repellents in a process referred to as negative chemotaxis. Bacteria exert negative chemotaxis to hydrogen peroxide and organic solvents such as alcohol. Upon exposure to repellants or intense light, ciliates and flagellates change the orientation of their swimming movement to avoid harm^3,4^. Cells of the cellular slime mold *Dictyostelium* can alter their movement when exposed to repellents^5^. Repellents in mammalian cells such as leukocytes and neuronal cells have also been identified. These repellents are known to play roles in axonal guidance^6^, resolution of inflammation^7^, gastrulation^8^, and metastasis^9^. Mobilization of cytoplasmic Ca^2+^ (Ca_i_^2+^) serves as an intracellular signal that is often observed when cells are exposed to repellents or dangers.

In a recent study, we developed a novel laser-based cell poration method to introduce foreign molecules into single cells that precisely injure the cell membrane by regulating the wound size^10^. The wound pores in the cell membrane promptly close by employing a wound repair system, which involves the recruitment of several repair proteins, such as annexin and actin^11^. The exact molecular mechanisms underlying wounding remain to be elucidated, although Ca^2+^ entry is believed to be the first trigger. Here, the present study is the first to demonstrate that when cells are locally wounded in the cell membrane by laserporation, they move away from the site of wounding. Furthermore, we demonstrated that cell migration can be manipulated by repeated wounding.

## Results and Discussion

### Cells escape the site of wounding

**Figure 1:**
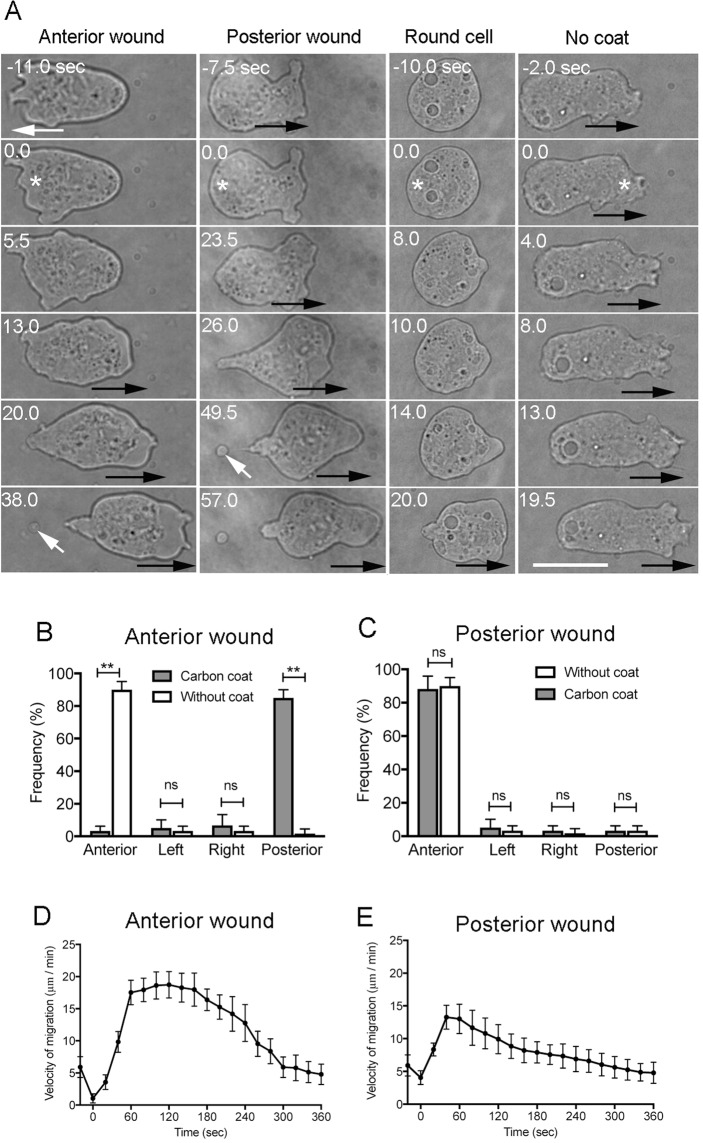
Cells escape the wounding site. (**A**) Cells were placed on a carbon-coated coverslip, and a laser beam was focused on a small local spot beneath a single cell under a TIRF microscope. When laserporation was applied at the anterior region of a migrating cell (asterisk), the cell stopped migration and retracted the anterior pseudopod (Anterior wound). Then, a new pseudopod protruded from the posterior region, and the cell migrated in the direction opposite to the wounding. On the other hand, when laserporation was applied at the posterior region of a migrating cell (asterisk), the cell did not change its direction of movement and quickly migrated in the same direction (Posterior wound). In many cases, a small cell debris was left behind the cell (white arrows), indicating that the wound membrane may be discarded as the cell advances. When laserporation was locally applied to an immobile round-shaped cell (asterisk), the cell began to migrate while extending a new pseudopod in the direction opposite from the wounding site (Round cell). For the control, laserporation was applied to cells on the coverslip without carbon coating (asterisk), and the cells did not show any response (No coat). Black arrows in each panel indicate the direction of cell migration. Bar, 10 µm. (**B**,**C**) Frequencies of cell migration in each direction (anterior, left, right, and posterior) after cells were wounded at the anterior or posterior regions on the coverslip, respectively, with or without carbon coating. Data are presented as mean ± SD (n = 60, each). **P ≤ 0.0001; ns, not significant, P > 0.05. (**D**,**E**) Time course of cell velocity after the cells were wounded at the anterior or posterior regions, respectively. Data are presented as mean ± SD (n = 20, each).

We used our novel laserporation method to create a local wound in the cell membrane of *Dictyostelium* cells. Cells were placed on a coverslip coated with carbon by vapor deposition, after which a laser beam was focused on a small local spot beneath a single cell using total internal reflection fluorescence (TIRF) microscopy. The energy absorbed by the carbon created a small pore in the cell membrane in contact with the carbon coat. The wound pores are promptly closed by the wound repair system within a few seconds^11^. Using the powerful laserpolation method, we examined the behavior of cells locally wounded at different sites. A typical polarized migrating cell contains one or two pseudopods at its anterior side that project outward to propel the cell forward. When laserporation was applied at the anterior region of a migrating cell (wound size of 1–1.5 µm in diameter), the cell stopped its movement and retracted the anterior pseudopod. Afterwards, a new pseudopod projected from the posterior region and the cell began to migrate towards the opposite direction (Fig. 1A, Anterior wound). On the other hand, when the laserporation was applied to the posterior region of a migrating cell, the cell did not change direction, although the velocity of cell migration was transiently increased (Fig. 1A, Posterior wound). When laserporation was locally applied in an immobile round-shaped cell, it began to migrate by extending a new pseudopod in the direction opposite to the wound site (Fig. 1A, Round cell). As a control, when the same strength of laser beam was applied to cells on coverslip without carbon coating, where no wound occurred (Fig. 1A, No coat), the cells did not show any response, suggesting that laser illumination does not induce the escape behavior. Figure 1B,C show the frequencies of cell migration in each direction after cells were wounded at the anterior or posterior sides on the coverslip, respectively, with or without carbon coating. Figure 1D,E show the changes in cell velocity over time after the cells were wounded at the anterior or the posterior regions, respectively. In both cases, the velocity of cell migration increased after a temporary decrease.

Previous studies have shown that when *Dictyostelium* cells were locally illuminated with microbeams of white light, low-intensity light favors the formation of pseudopods at the irradiated parts of the cells, whereas strong illumination suppresses the extension of pseudopods, thereby suggesting that the cells can respond to local continuous light^12^. However, the laser beam used in the present study illuminated the cells for an extremely short period (10 ms), which did not affect cell behavior. Therefore, we concluded that local wounding induced the escape behavior of cells.

### Local wounds can manipulate cell migration

**Figure 2:**
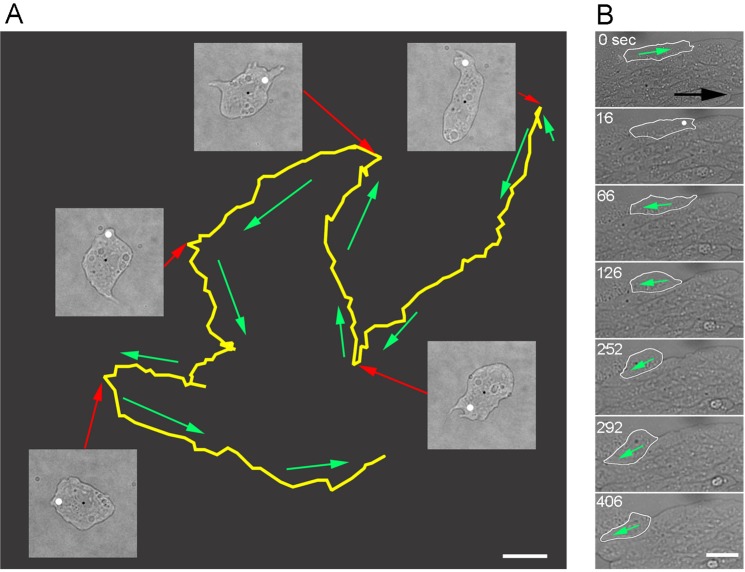
Local wounds can manipulate cell migration. (**A**) Typical trajectory of cell migration after being subjected to repeated multiple local wounding. Laserporation was applied for five times at the anterior regions of the migrating cell. Yellow line represents the trajectory of cell centroid. Five bright field images are shown when the laserporation was applied (white spots). Red arrows indicate the positions in the trajectory after cell wounding. Green arrows indicate the direction of cell migration. The direction of movement changed each time the cell was wounded. (**B**) In the aggregation stream, laserporation was applied at the anterior region of a single cell (white spot). The wounded cell (outlined with white line) migrated in the opposite direction after wounding. Note that other cells migrated towards the right direction (black arrow). Bar, 10 µm.

We previously showed that *Dictyostelium* cells can survive multiple wounding^11^. Next, we examined whether the polarity of cell migration can be manipulated by the introduction of repeated local wounds. To explore the above hypothesis, we applied laserporation multiple times to a single cell and followed the trajectory of cell migration by microscopy. Figure 2A shows a representative experiment (Supplementary Video S1). A single cell was monitored, and laserporation was applied five times at the anterior region of the cell. The cell was observed to change polarity after each laserporation event. Similar results were observed in 15 cells. Therefore, the laserpolation method was successfully employed to manipulate the direction of cell migration.

The development of *Dictyostelium* is characterized by the formation of multicellular aggregates. The cells migrate towards aggregation centers by chemotaxis to form aggregation streams. All cells in a stream migrate in the direction towards the center while being attached with each other side-by-side and end-to-end. When laserporation was applied to the anterior region of a single cell, the cell stopped migrating and moved in the opposite direction (Fig. 2B and Supplementary Video S2). Similar results were confirmed in 30 cells. Therefore, wound-induced reversal occurs even in multicellular environments and appears to surmount cell-cell adhesions or external chemotactic signals.

### Signals for cell escape

**Figure 3:**
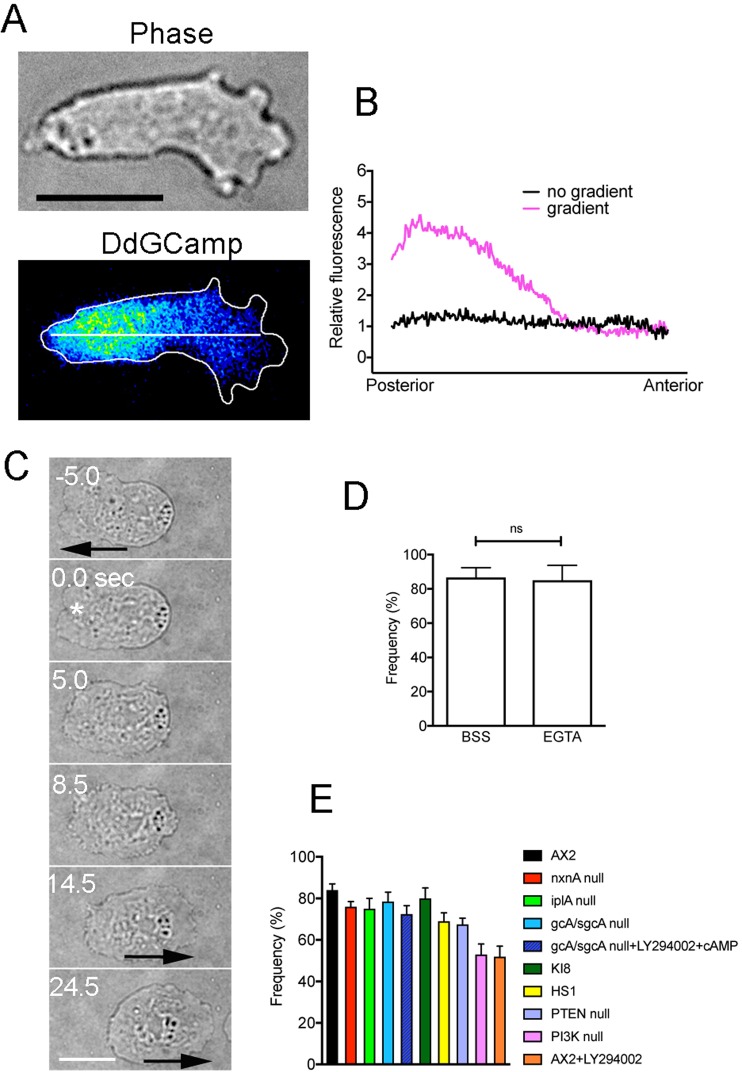
Signals for the escape behavior of cells. Supplementary Tables cells expressing Dd-GCaMP6s were observed by TIRF microscopy. (**A**) typical cell showed a shallow decreasing Ca_i_^2+^ gradient from the posterior to the anterior regions. However, the gradient was observed in only 20% of examined cells (n = 100). (**B**) Quantitative analysis of fluorescence intensities along the long axis of migrating cells as shown as the white line in Panel A. Two curves were derived from averaged curves of 17 cells with and without the gradient. (**C**) Typical cell behavior after wounding at the anterior region (asterisk) in the presence of 5 mM EGTA. Arrows indicate the direction of cell migration. (**D**) Frequency of the reversal after cells were wounded at the anterior regions in the presence of BSS and EGTA. Data are presented as mean ± SD (n = 30, each). (**E**) Frequencies of the opposite migration of mutant or wild-type cells in the presence of inhibitors after local wound. Data are presented as mean ± SD (n = 70, each). Detailed description of mutants and inhibitors are described in Supplementary Table S1. Bars, 10 µm.

The increase in cytosolic Ca^2+^ (Ca_i_^2+^) levels is often used as a signal for cells to avoid repellents or dangers. Transient increases in Ca_i_^2+^ levels have been reported in various species when cells change their polarity^13–18^. In addition, a Ca_i_^2+^ gradient has been indicated to participate in cell migration^19–23^. In addition, using the Ca_i_^2+^ sensor Fura-2, we previously observed a decreasing Ca_i_^2+^ gradient from the posterior to anterior regions in actively migrating *Dictyostelium* cells, although such a gradient was limited to a small population of cells^24^. Here, using cells expressing Dd-GCaMP6s, a sensor of Ca^2+^, we confirmed the presence of a decreasing Ca_i_^2+^ gradient from the posterior to anterior regions of polarized migrating cells (Fig. 3A,B). However, the gradient was observed in only 20% of examined cells (n = 100), the exact reason of which remains unknown. In addition, an increase in the total Ca_i_^2+^ levels was not observed when the cells changed polarity during normal migration (without wounds), although such a temporal increase in Ca_i_^2+^ levels has been reported in eosinophils^19^.

Despite the above observations, Ca^2+^ influx from the wound pore, which was previously reported in cells expressing Dd-GCaMP6s^11^, was presumed to contribute to the wound-induced escape behavior. Next, we examined whether the entry of Ca^2+^ into the cytosol is essential for the reverse behavior by incubating the cells in EGTA, a Ca^2+^ chelator. In the presence of EGTA, Ca_i_^2+^ does not increase after wounding as shown previously^11^. Contrary to the expected results, when wounded at the anterior region, the cells retracted the anterior pseudopods, extended a new pseudopod in the opposite direction, and then migrated in the opposite direction (Fig. 3D). Similar results were confirmed in 30 cells.

We previously showed that mutant cells deficient in inositol 1,4,5-trisphosphate receptor-like protein (iplA), which is involved in the release of Ca^2+^ from intracellular stores via a calcium-induced calcium release (CICR), significantly inhibited the increase in Ca_i_^2+^ levels after wounding^11^. IplA null cells did not show any defects in the reversal behavior after wounding (Fig. 3E). Together, we concluded that neither Ca^2+^ influx nor the global increase in Ca_i_^2+^ levels contributed to the escape behavior.

We then sought to determine the signal that contributes to the escape behavior of cells. Many types of cells, including *Dictyostelium*, are known to respond to electric fields by directional migration. Reversal of field polarity causes a reversal in the direction of *Dictyostelium* migration, which requires Ca^2+^ influx^25^, whereas a recent study showed that the reversal does not occur when both cGMP production and PI3Ks are inhibited^26^. Furthermore, *Dictyostelium* cells showed a similar reversal behavior when a chemorepellent was applied to the rear side^5,27^. PI3K-null cells and phospholipase C-null cells did not show the reversal, suggesting that the repellent induced dominant phosphatidylinositol-3,4,5-trisphosphate (PI(3,4,5)PI_3_) signaling at the posterior end of the cell, which caused the cell to move away from the repellent^27^. To examine whether these signals are involved in the wound-induced reversal, relevant mutants and inhibitors (PI3K-null cells, gcA/sgcA-null cells, KI8, PTEN-null cells, myosin II-null cells, and LY294002) were evaluated (the detailed descriptions of the mutants are described in Supplementary Table S1). All the examined cells exhibited partially round-shaped and less polarized. When laserporation was applied at the one side, they began to migrate by extending a new pseudopod in the opposite direction to the wound site. We confirmed this escape reaction in at least 70 cells for each case (Fig. 3E). Supplementary Video S3 shows a representative movie of PI3K-null cells, which are the most less-polarized in the examined mutants.

The above results indicated that well-known diffusive signals and lipid signals do not contribute to the escape behavior. Presumably, the creation of a pore in the cell membrane could be crucial for the escape behavior.

Membrane tension has been proposed to regulate cell motility and influence the anterior-posterior axis by coordinating with cytoskeleton^28–30^. We previously observed a temporal expansion of the pore after wounding^11^. The observed local decrease in cell membrane tension, which generates a new gradient of membrane tension along the cell body, could induce the rearrangement of actin cytoskeleton, retraction of old pseudopods, and extension of a new pseudopod on the opposite side of the cell. The present study provided insights into the mechanisms by which cells establish their polarity.

Finally, we showed that cells move away from the site of wounding. If any treatment fails to kill cancer cells but causes recoverable wounding, the motility and invasive activities of cancer cells might be induced. We believe that our results will contribute to the applied medical field.

## Methods

### Cell culture

*Dictyostelium discoideum* cells (AX2) were cultured in plastic dishes at 22 °C in HL5 medium (1.3% bacteriological peptone, 0.75% yeast extract, 85.5 mM D-glucose, 3.5 mM Na_2_HPO_4_. 12H_2_O, and 3.5 mM KH_2_PO_4_, pH 6.3) as previously described^31^. The cells were transformed with extrachromosomal vectors for the expression of Dd-GCaMP6s by electroporation as previously described^32^. The transformed cells were selected in HL5 medium supplemented with 10 µg/ml G418 (Wako, Japan). For the wounding experiments, HL5 medium was replaced with BSS (3 mM CaCl_2_, 10 mM KCl, 10 mM NaCl, and 3 mM MES, pH 6.3), after which the cells were incubated in the same solution for 5 to 6 h. To obtain cells in the aggregation stage, cells were incubated at 10.5 °C overnight. Several mutant cells were additionally cultured by the same method. KI-8 cells were cultured on a 5LP plate (0.5% lactose, 0.5% proteose peptone, and 2% agar) with *Escherichia coli* (B/r) at 22 °C.

### Carbon coating and chamber preparation

The surface of the coverslip in a glass-bottom chamber was coated with carbon by vapor deposition using a vacuum evaporator (JEOL, JEE-400). The thickness of the carbon layer was approximately 20 nm. To make the surface hydrophilic, the surface of the carbon-coated coverslip was activated by plasma treatment. The chamber was sterilized with 70% ethanol and dried when necessary. The cells were settled on the surface of the carbon-coated coverslip and then slightly compressed using an agarose block (1.5%, dissolved in BSS, 1-mm thick) to observe the ventral cell surface^33^. Under these conditions, the cells can migrate by extending pseudopods^34^.

### Laserporation

Fluorescence images of cells expressing GFP-proteins were observed under a total internal reflection fluorescence microscope (TIRF, based on IX71 microscope, Olympus) as previously described^35^. For laserporation, a nanosecond-pulsed laser (FDSS532-Q, CryLas) beam was directed towards each sample using a dichroic mirror through the TIRF microscope as previously described^10^. The laser beam (wavelength, 532 nm) was operated with 15 mW output power and pulse width of 1 ns and attenuated to 1/300 via passage through several neutral density filters. A 60x (PLAPON60XOTIRFM, Olympus, NA = 1.45) objective was used to focus the laser beam on the surface of the carbon-coat. The x and y coordinates of the laser spot were fixed in the center of the microscopic field. The position of the cells was moved using a piezo-motorized x-y stage on the microscope. The duration of the laser beam application was set to 4 to 40 ms and controlled using shutter. The wound size was controlled by changing the laser power or the size of pinhole, which was inserted in the path between the laser and dichroic mirror. Time-lapse fluorescence images were acquired with 50–100 ms exposure times at 200–500-ms intervals using a cooled CCD camera (Orca ER, Hamamatsu Photonics).

### Image analysis

The acquired images were analyzed using Image J software (http://rsbweb.nih.gov/ij/). Cell velocity was determined from the centroid after obtaining the cell outline. Quantitative profiles of the fluorescence intensities of GCaMP6s along the long axis of migrating cells were analyzed with Image J. The relative fluorescence intensities were derived from the averaged curves of 17 cells after subtracting each value from the background value. Graphs were created using GraphPad Prism 7 (GraphPad Inc., USA) based on the calculations using Microsoft Excel.

### Statistical analysis

Statistical analysis was performed using GraphPad Prism 7. Data are presented as mean ± SD and analyzed by one-way ANOVA with Tukey’s multiple comparison test.

## Supplementary information

Supplementary Video S1

Supplementary Video S2

Supplementary Video S3

Supplementary Information

## Data Availability

All relevant data are available from the authors on reasonable request.
