# Supplementary material for: Manipulation of cell migration by laserporation-induced local wounding
Source: Sci Rep. 2019 Mar 12;9:4291. doi: 10.1038/s41598-019-39678-1 (PMC6414676; doi:10.1038/s41598-019-39678-1)
Supplement: Supplementary file 4 — Supplementary Information [file 41598_2019_39678_MOESM4_ESM.docx]

Supplementary Information for

**Manipulation of cell migration by laserporation-induced local wounding**

Mst. Shaela Pervin and Shigehiko Yumura

**Supplementary Table**

**Supplementary Table S1 Description of mutant cells and inhibitors used in the present study**

| Mutants or inhibitors | Description |
| --- | --- |
| AX2 | Wild-type cells. |
| nxnA-null | Annexin C1 null cells. Annexin accumulates at the wound site to promote repair ^11^. |
| iplA-null | IplA (Inositol 1,4,5-trisphosphate receptor-like protein)-null cells. IplA protein is involved in the release of Ca^2+^ from intracellular Ca^2+^ stores ^25^. |
| gcA/sgcA-null | Guanylyl cyclase A (gcA) and soluble guanylyl cyclase (sgcA)-null cells. These mutant cells do not exhibit guanylate cyclase activity ^36^. |
| gcA /sgcA-null + LY294002 + cAMP | In the presence of 1 µM cAMP and 60 µM LY294002, the mutant cells exhibit electrotaxis towards the anode, whereas wild-type cells move towards the cathode ^26^. LY294002 is an inhibitor of PI3K. |
| KI-8 | Generation of chemotaxis-deficient mutants by chemical mutagenesis. Mutants have no guanylate cyclase activity and exhibit electrotaxis towards the anode, whereas wild-type cells move towards the cathode ^26^. |
| HS1 | Myosin II heavy chain-null cells. Myosin II stabilizes cell polarity ^37^. |
| PTEN-null | PTEN (phosphatidylinositol-3,4,5-trisphosphate 3-phosphatase)-null cells. PTEN phosphatase regulates PI3K signaling by dephosphorylating PIP3. Furthermore, PTEN contributes to cell polarity ^38^. |
| pi3k-null | Sextuple phosphoinositide 3-kinase-null cells. These mutant cells do not show the reversal when a chemorepellent is applied to the posterior region of a migrating cell ^39^. |
| LY294002 | AX2 cells in the presence of PI3K inhibitor. |

**Supplementary Video Legends**

**Supplementary Video S1**

**Manipulation of cell migration by multiple wounds**

Actual movement of the cell shown in Figure 2A at 10-s intervals. White dots indicate the wound position. Laserporation was applied five times.

**Supplementary Video S2**

**Wound-induced reversal in the aggregation stream**

Actual movement of the cell shown in Figure 2B. In the aggregation stream, laserporation was applied at the anterior region of the single cell (white dot). The wounded cell migrated in the direction opposite to the site of wounding. Note that other cells migrated towards the right.

**Supplementary Video S3**

**Wound-induced escape behavior of a PI3K-null cell**

When locally wounded (white dot), the PI3K-null cell began to migrate by extending a new pseudopod in the opposite direction to the wound site.
